# Microbial Ecosystems in Movile Cave: An Environment of Extreme Life

**DOI:** 10.3390/life13112120

**Published:** 2023-10-26

**Authors:** Joost W. Aerts, Serban M. Sarbu, Traian Brad, Pascale Ehrenfreund, Hans V. Westerhoff

**Affiliations:** 1 Molecular Cell Biology, A-LIFE, 01-E-57, Faculty of Science, VU University Amsterdam, Van der Boechorstraat 3, 1081 BT Amsterdam, The Netherlands; 2 “Emil Racoviţă” Institute of Speleology, Str. Frumoasă 31, 010986 Bucharest, Romania; 3 Department of Biological Sciences, California State University, Chico, CA 95929, USA; 4 “Emil Racoviţă” Institute of Speleology, Clinicilor 5-7, 400006 Cluj-Napoca, Romania; traian.brad@ubbcluj.ro; 5 Laboratory for Astrophysics, Leiden Observatory, Leiden University, 2333 RA Leiden, The Netherlands; 6 Space Policy Institute, George Washington University, Washington, DC 20052, USA; 7 Synthetic Systems Biology and Nuclear Organization, Swammerdam Institute for Life Sciences, University of Amsterdam, 1098 XH Amsterdam, The Netherlands; 8 School of Biological Sciences, Faculty of Biology, Medicine and Health, University of Manchester, Manchester M13 9PL, UK; 9 Stellenbosch Institute for Advanced Study, Stellenbosch 7600, South Africa

**Keywords:** Movile Cave, microbial communities, 16S, minerals, chemosynthesis, extremophiles

## Abstract

Movile Cave, situated in Romania close to the Black Sea, constitutes a distinct and challenging environment for life. Its partially submerged ecosystem depends on chemolithotrophic processes for its energetics, which are fed by a continuous hypogenic inflow of mesothermal waters rich in reduced chemicals such as hydrogen sulfide and methane. We sampled a variety of cave sublocations over the course of three years. Furthermore, in a microcosm experiment, minerals were incubated in the cave waters for one year. Both endemic cave samples and extracts from the minerals were subjected to 16S rRNA amplicon sequencing. The sequence data show specific community profiles in the different subenvironments, indicating that specialized prokaryotic communities inhabit the different zones in the cave. Already after one year, the different incubated minerals had been colonized by specific microbial communities, indicating that microbes in Movile Cave can adapt in a relatively short timescale to environmental opportunities in terms of energy and nutrients. Life can thrive, diversify and adapt in remote and isolated subterranean environments such as Movile Cave.

## 1. Introduction

Terrestrial extreme environments are widely used to study the limits of life [1,2]. They may also serve as analogue environments for studying the potential for the habitation of other planets [2,3]. Due to the hostile surface conditions on many planetary bodies, the search for life beyond Earth should perhaps focus on the subsurface [2,3]. But could such a subsurface environment be capable of sustaining its own biosphere without a continuous influx of living organisms from the outside world? And if so, would its life be sparse or abundant? And would it be highly specialized or diverse? If diverse, would the diversity be predictable?

One subsurface environment that might serve as an analogue of strongholds for life is Movile Cave (Romania), located near the shores of the Black Sea. 

A unique groundwater ecosystem exists in Movile Cave, in which microbial communities serve as primary biomass producers [1] (in Section 4.2, we will further discuss how these chemolithotrophic organisms may survive the cave environments).

**Figure 1:**
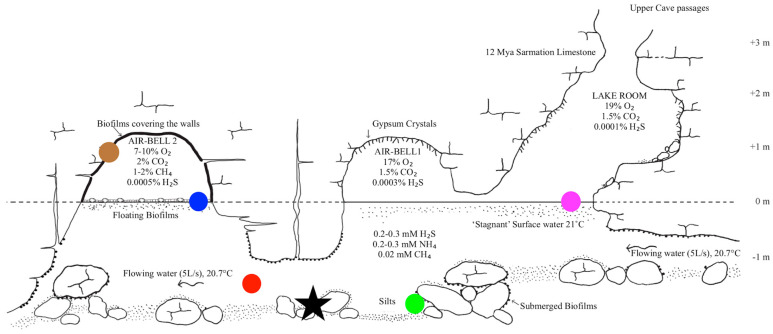
Cross-section of the lower, partially submerged level of the Movile Cave system. Modified after [13]. Samples were collected from different sublocations, which are indicated by colored circles: from the surface waters of the Lake Room (pink); from floating biofilms at the water surface in Air-Bell 2 (blue) as well as from biofilms on the ‘dry’ walls (brown) that form the dome of Air-Bell 2; from deeper, oxygen-poor waters between Air-Bell 1 and Air-Bell 2 (red); and from biofilms attached to rocks at the bottom of the submerged section between Air-Bells 1 and 2 (green). The location where the microcosm experiment was executed is shown as a black star. The vertical scale is centered on the water–atmosphere interface (at what was defined as ‘0 m’). The horizontal scale was adapted for this figure (real length from left to right is approximately 15 m).

Situated 18 m below the surface, the cave consists of a 200 m long upper dry passage that ends at a small underground lake and provides access to a 40 m long, partially submerged lower cave level (Figure 1). It was discovered in 1986 during construction work and since then can only be entered via a vertical (sealed-off) shaft descending from the surface. For the last 5.5 million years, significant input of meteoritic water and organics from the surface has been prevented by impermeable layers of clay and loess that cover the limestone in which the cave was formed [4]. The absence from the waters of Movile Cave of radioactive cesium and strontium isotopes that were widespread in the region due to the Chernobyl disaster in 1986 indicated that the cave is essentially sealed off from the surface [1].

Mesothermal, sulfidic groundwater (up to 1 mM in H_2_S) originating from an artesian sulfidic aquifer underlying the region enters at the bottom of the cave’s lower passages. This results in a constant flow occurring tens of centimeters to 1 m below the water surface at flow rates of about 5 L/s [5], which causes a continuous slow-motion movement of the surface waters. The ecosystem relies on biomass production by prokaryotes that derive metabolic energy from the oxidation of hydrogen sulfide (H_2_S), methane (CH_4_) and ammonium (NH_4_^+^) and assimilate carbon from the cave’s atmospheric methane and carbon dioxide [1,6,7]. Common electron acceptors are dioxygen (O_2_), nitrate (NO_3_), sulfate (SO_4_^2−^) and ferric iron (Fe^3+^). The waters of the cave are neutral in pH due to buffering by carbonates derived from the limestone bedrock [5].

The atmospheric composition in the cave differs from that outside, with elevated CO_2_ (1–2%) and lower O_2_ concentrations (19%). Separated by submerged passages from the rest of the cave’s dry upper passages (Figure 1), the Air-Bells (especially Air-Bell 2) differ even more, with high levels of methane and carbon dioxide and low levels of oxygen (7–10%). The surface waters below the Air-Bells are for a large part covered by thick biofilms, while the surface waters in the ‘Lake-Room’ display only a thin, loose, white surface layer. Oxygen levels decline rapidly to anoxic or microaerobic (see below) conditions underneath the water surface in the Lake Room; deeper water layers in Movile Cave may contain no dissolved oxygen [5,8].

The bedrock surrounding the cave may provide additional elements for the microbial life in the cave [5,8]. Therefore, targeting mineral-rich outcrops as potential habitats for underground microbial life seems a valid approach in the search for extreme life. Respiration using alternative electron acceptors, which can be provided by minerals, reduces the need for oxygen. Minerals also offer protection, sites for bacterial adhesion and surfaces where the polymerization of smaller molecules can occur [9]. Mineral microcosm experiments confirmed that microbial colonization is correlated with mineral types [10,11,12].

In this paper we address several questions. Would minerals also affect the microbial ecostructure in a microcosm experiment conducted in a specialized and extreme environment like Movile Cave? Would the microbial life colonizing the microcosms be a monoculture or a community of organisms not dominated by a single fittest species? Would multiple communities exist more or less at random or in a deterministic and potentially predictable manner? Would we find the same microbial profile on all minerals, or would each mineral develop its own? In search of answers, biological samples were collected from preexisting Movile Cave sublocations as well as from added microcosms and analyzed for microbial composition using a course-grained method: 16S rRNA Illumina sequencing. We report a substantial diversity of microbial life endemic to Movile Cave, with community structures specific both to its subenvironments and to the added microcosms and which are largely stable over periods of at least three years.

## 2. Materials and Methods

### 2.1. Visits to Movile Cave

The cave was visited and sampled three times over the course of three successive years (2015–2017) during August, May and May, respectively. An experienced cave diver joined the expedition every year to reach and collect samples from areas that are separated from the Lake Room by flooded passages.

### 2.2. Sample Collection

To collect the microbial species from cave water, a syringe was used to force approximately 0.20 L samples through sterile 0.2 μm pore-size filters (Whatman FP30/0.2 CA-S) encased in filter holders. Filters were afterwards stored in sterile Greiner tubes. The deep-water samples were collected in sterile Greiner tubes in the deeper water layers and then transported to the surface with the caps screwed on. Surface-water samples were collected directly from the surface water in the Lake Room (for the sampling sites, see Figure 1). Samples from the submerged biofilms were collected as follows: A fragment of a rock from the bottom of the submerged passages on which these biofilms grow was transported to the surface. There, using sterile spatulas, a sample of the biofilm was scraped off the rock and stored in a sterile Greiner tube. Samples from the floating biofilms in Air-Bell 2 were collected by directly forcing a portion of the floating biofilm into a 50 mL sterile Greiner tube; the tubes were subsequently transported through the submerged passages to the Lake Room. Biofilms from the dry walls of the dome in Air-Bell 2 were collected in sterile Greiner tubes and subsequently transported with the screwcap closed through the submerged passages to the Lake Room. Until further analysis, samples were kept refrigerated at 4 °C as much as possible. During transport by airplane, the samples were wrapped in aluminum foil to limit quick and extreme temperature fluctuations. After transport, samples were kept refrigerated at 4 °C until DNA extraction, which took place within 2 days.

### 2.3. Site Measurements

Dissolved oxygen concentration, temperature and salinity of the water were determined using a GMH 3410 CE digital multitool (Greisinger Electronic, Regenstauf, Germany). Oxygen in the atmosphere was determined using an oxygen probe. The pH values of the water samples were determined using pH paper.

### 2.4. Microcosm Experiment

Obsidian was purchased from Terra Incognita (Amsterdam, The Netherlands) and was of a black variety. All other minerals were purchased from Excalibur Mineral Corporation (Charlottesville, VA, USA) and selected for their purity as listed on the company website. All minerals were ground to obtain particles of 100–200 μm using an automatic grinder and sieves kindly provided by the Vrije Universiteit Amsterdam Geology Department. Ground minerals were sterilized by heating in an oven at 140 °C for 24 h and were subsequently placed in semipermeable nylon bags with a pore size of 10 μm. Each bag contained approximately 25 g of mineral and bags for every mineral type were prepared in triplicate. Of this same mineral batch, material was kept aside to serve as a blank control for the minerals. DNA analyses of these mineral blanks were subtracted from the incubated mineral DNA analysis.

The nylon bags containing the minerals were stored in a self-constructed PVC device with 15 different compartments for 3 mineral bags each. Slits in the PVC construction allowed the free flow of water through the compartments, which should suffice to equilibrate pH and temperature to the same extent as elsewhere in the cave water. Thick stainless-steel screws and screwcaps held the compartments together, and two PVC plates covered the top and bottom of the five pillars (Figure S1). Mineral bags were allocated at random to compartments. The device was sterilized by autoclave prior to insertion of the mineral bags. The device containing the mineral bags was transported in a sterile bag to the cave. In the cave the device was placed in a side passage of the submerged section between Air-Bell 1 and Air-Bell 2 at the bottom of the submerged passage and was left there undisturbed for 1 year. It was retrieved by bringing it to the Lake Room, where the bags were cut free and placed in sterile Greiner tubes before transportation to the laboratory where the bags were cut open and the minerals were subjected to DNA extraction.

### 2.5. DNA Extraction

Prior to DNA extraction, filters containing microbial load from water samples were cut into small pieces and placed in the tube for DNA extraction. Blank filters were subjected to the same procedure to serve as controls. Biofilm samples were vortexed thoroughly to homogenize the samples before 0.5 g of the biomass was placed in the extraction tube. Procedural controls were included for all the extraction rounds (i.e., extraction tube was opened, but no material was placed in it). Positive controls were also included during the extraction process. For the microcosm experiment 1 g of each mineral replicate was used for extraction. The same amount of material was used for the blank mineral extractions.

The DNeasy PowerSoil extraction kit (Qiagen, Hilden, Germany) was used for the DNA extraction of the samples, following the manufacturer’s protocol. All handlings of the samples and controls during the extraction process were conducted in a UV3 HEPA PCR workstation (UVP, Upland, CA, USA), equipped with a HEPA filter and a UV illuminator to prevent extraneous DNA contamination. DNA concentrations of the extracts were determined using a Quant-iT high-sensitivity DNA assay kit and a Qubit^®^ fluorimeter (Invitrogen, Carlsbad, CA, USA). DNA extracts were stored at −20 °C until further processing.

### 2.6. Illumina 16S Amplicon Sequencing

All DNA extracts were diluted to a concentration of 0.1 ng/μL prior to PCR amplification. PCR reactions were performed in triplicate using Phusion Green Hot Start II High-Fidelity DNA Polymerase (Thermo Fisher Scientific, Stockholm, Sweden). We targeted the V3-V4 region of the 16S rRNA gene, using the V3 forward primer S-D-Bact-0341-b-S-17, 5′-CCTACGGGNGGCWGCAG-3 [14] and the V4 reverse primer S-D-Bact-0785- a-A-21, 5′-GACTACHVGGGTATCTAATCC-3′ [15], giving rise to ~430 bp dsDNA fragments.

The primers were dual barcoded and were compatible with Illumina sequencing platforms as described previously [16]. Performance of the PCR reaction was checked by running incorporated positive and negative controls from each triplicate plate on 1.5% (*w*/*v*) agarose gels. Triplicate PCR products were combined, and each combined triplicate sample was purified using SPRI beads (Agencourt^®^ AMPure^®^ XP, Beckman Coulter, CA, USA). The DNA concentration in the purified samples was determined as described above. Samples were diluted to identical concentrations of 2 ng/μL prior to pooling the diluted PCR products together in equal volumes (10 μL) in one composite sample (including positive and negative controls).

The composite samples were paired-end sequenced at the Vrije Universiteit Amsterdam Medical Center (Amsterdam, The Netherlands) on a MiSeq Desktop Sequencer with a 600-cycle MiSeq Reagent Kit v3 (Illumina) according to manufacturer’s instructions. High-throughput sequencing raw data were demultiplexed using bcl2fastq software version 1.8.4 (Illumina) and primers were trimmed using Cutadapt [17]. De-multiplexed samples were further processed using a modified version of the Brazilian Microbiome Project 16S profiling analysis pipeline [18]. Paired-end reads were joined using PANDAseq [19] allowing for a minimum overlap of 30 nucleotides between the forward and reverse reads, a minimum sequence length of 285 whilst no mismatches in the primer region were allowed. PANDASeq addresses mismatches in overlapping regions by selecting the nucleotide with the best sequencer-assigned quality score. Because PANDAseq incorporates a base quality filter during read assembling, the threshold for consecutive high-quality bases per read was set to zero. Metadata and demultiplexed samples were merged using add_qiime_labels.py [20] and sequence headers were changed using bmp-Qiime2Uparse.pl [18]. UPARSE was used to dereplicate, to filter chimeras, to discard OTUs detected less than 2 times, and for OTU clustering at 97% similarity [21,22]. The OTU taxonomy was assigned using the UCLUST algorithm [21] on QIIME [20] using SILVA compatible taxonomy mapping files (Silva database release 128) [23,24] and aligned using align_seqs.py in QIIME [25]. Taxonomy was manually curated and refined up to genus level based on 97% similarity of reference sequences, the reference tree being calculated using the make_phylogeny.py script in QIIME [26]. We generated a BIOM file using make_otu_table.py on QIIME [20]. Prior to further analysis we produced an OTU table and a taxonomy table using BIOM scripts [27]. The OTUs also detected in negative or procedural controls were manually removed from the dataset. Lastly, a trimming step removed sequences that made up less than 0.05% of the dataset in order to reduce background noise [28].

This Targeted Locus Study project has been deposited at DDBJ/ENA/GenBank under the accession KERY00000000, KERZ00000000. The version described in this paper is the first version, KERY01000000, KERZ01000000. Separate Bioprojects were created for the microcosm experiment (PRJNA703821) and the experiment focusing on the endemic communities in the cave (PRJNA702640).

### 2.7. Data Analysis and Processing

After removal of contaminating sequences in the controls, removal of singletons and performing a trimming step (see below), the dataset composed of the samples from the various sublocations in the cave consisted of 2,068,287 sequence reads and a total of 1378 OTUs (Operational Taxonomic Units). 

For most analyses, a rarefied dataset was used (35,588 sequence reads per sample), which contained 1377 OTUs in total (we lost 1 OTU in the rarefaction process).

For the mineral microcosm dataset, besides performing the same steps as described above for the environmental samples from the cave, sequences found in mineral blanks (non-incubated) were subtracted from the dataset. After these steps, the dataset was composed of 2,869,940 sequence reads with a total of 802 OTUs. After rarefaction (either to 19,767 or to 7600 sequence reads) the dataset still contained 802 OTUs in total.

The principal coordinates analyses were produced with the function beta_diversity_through_plots.py on QIIME using weighted unifrac metrics on a rarefied dataset. The Phyloseq package (https://joey711.github.io/phyloseq/ (accessed on 23 October 2023)) was used for further data analysis and visualization by importing the BIOM table and metadata into the R-Studio environment. For alpha diversity analysis and visualization we used the plot richness function from the Phyloseq package in RStudio (https://posit.co/download/rstudio-desktop/ (accessed on 23 October 2023)).

For Chao1 alpha diversity the following formula was used: S_p_ = S_0_ + a_1_ · a_1_/(2 · a_2_) · ((N − 1)/N) in which S_p_ is the extrapolated richness in the species pool (Chao1), S_0_ is the observed number of OTUs in the collection, a_1_ and a_2_ are the number of species occurring only in one or in two sites, respectively, in the collection and N is the number of sites in the collection. For Shannon index *H* the following formula was used: *H = -∑p_i_* · *ln(p_i_)*, where *p_i_* is the relative abundance of species *i*. The adonis function and the betadisper function from the vegan package (https://www.researchgate.net/publication/313502495_Vegan_Community_Ecology_Package (accessed on 23 October 2023)) were used in R-studio for the *adonis* tests (Permutational Multivariate Analysis of Variance Using Distance Matrices) and homogeneity of dispersion tests, respectively. Weighted unifrac distance metrics were used for these analyses. The betadisper procedure is often used as an indirect measure of beta diversity (see the Vegan documentation). The outcome of the betadisper function was tested for significance using the function permutest from the Vegan package as described in the Vegan documentation (page 195), using sample location (for the endemic communities in Movile Cave), mineral type and absence/presence data of the mineralogical components (as derived from the minerals’ chemical formula) as explanatory variables.

## 3. Results

In the Supplementary Materials, our results are discussed in more detail (Section S1). Here we discuss the most important results.

### 3.1. Environmental Measurements and Observations in the Cave

Biofilms or veils (the basis of these layers is not necessarily biological) were present at the water surface and covered the walls in numerous locations. The air smelled of rotten eggs indicating elevated levels of H_2_S [1]. Several environmental measurements were taken (in 2017 and only in the Lake Room, Figure 1): The pH value of the water was 7.5. The water temperature was 21 °C and the electroconductivity (EC) was 0.14 S/m, which translates roughly to a salinity of 0.5% according to the manufacturer’s protocol. Oxygen concentration in the atmosphere of the Lake Room was 19% (as opposed to 21% at the surface above the cave). Riess et al. [8] analyzed the change in the concentration of O_2_ in the water column and found traces of oxygen up to a maximum depth of 0.8 mm, while the water below was concluded to be anoxic.

When we determined the oxygen concentration in the water column of the Lake Room using an oxygen probe (Figure S2), oxygen levels steadily declined with depth, with the water never becoming fully anoxic. This is more consistent with the reduction in oxygen concentration being caused by life processes, as it should then stop near the smallest Michaelis constant for oxygen, which may be close to 5 microM [29], corresponding to the 0.15 mg/L we observed 1 m below the water surface. 

A substantial influx of H_2_S and its reduction in molecular oxygen should, however, move the aerobic–anaerobic interface upwards. We note that these mechanisms should cause the oxygen gradient to depend on the local microbial ecologies and geologies (and vice versa), so that the oxygen gradient may well differ between the various locations in the cave.

### 3.2. Endemic Microbial Ecology in Movile Cave: Diversity within and between the Sublocations

**Figure 2:**
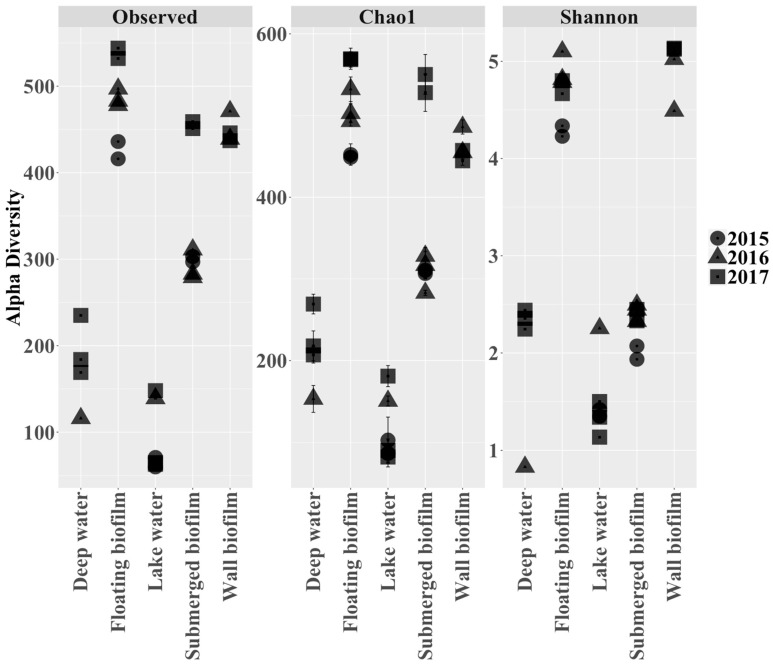
Alpha (within sampling site) diversity metrics per sample expressed as the observed number of species (‘Observed’), the predicted number of species based on sequencing depth (‘Chao1′) or as the Shannon index, which indicates how evenly the species are distributed (‘Shannon’). Alpha diversity measurements are shown for all 28 samples taken at 5 locations in the cave (4 deep water; 7 floating biofilm; 6 lake water; 7 submerged biofilm; 4 wall biofilm) over 3 subsequent years (indicated by different shapes). Error bars for the Chao1 index show the standard error for the estimated richness in the model. All metrics were calculated by using the estimate richness function in the Phyloseq package in R studio). Samples were rarefied to an equal depth of 35,588 reads per sample.

Biological samples taken from a variety of sublocations over the course of three successive years (2015–2017) (Figure 1) were analyzed using Illumina 16S rRNA sequencing. A total of 1378 unique OTUs were detected in the final 16S rRNA library. Alpha (i.e., within sampling site) diversity assessment of a rarefied dataset (35,588 reads per sample included) showed that the number of observed OTUs per sample ranged from around 100 ‘species’ (OTUs) to more than 500 (Figure 2). The more diverse samples came from the sublocations containing biofilms (submerged biofilm, floating biofilm, wall biofilm) (Figure 1). The less diverse samples were water samples (deep water and lake water) (Figure 1). Shannon indices reflected that floating-biofilm and wall-biofilm communities had a more even distribution (i.e., many species contributing with similar abundances), while communities in the cave waters and in the submerged biofilm had skewed distributions with a single or a few species dominating.

The distribution of the 50 most abundant prospective families is shown in Figure S3. Taxa belonging to unknown/uncultured families were almost exclusively found in the biofilm samples (see Supplementary Materials for the complete OTU table; Table S1). The observed distribution leads us to conclude that there is a substantial difference in microbial communities between sublocations, i.e., there is a high beta diversity if we compare the different sublocations (which are indicated by the capital letters below the bars, i.e., DW (deep water), FB (floating biofilm), LW (lake water), SB (submerged biofilm), and WB (wall biofilm), as if they are different ecosystems. Part of this observed beta diversity is caused by varying abundances of ‘common species’, i.e., ‘species’ present throughout the different cave areas (see Figure S4 for percentages of species found to be shared between the cave’s subenvironments). However, especially for the different biofilm samples, a large number of species was exclusively detected in that specific subenvironment (these are called ‘unique’ in Table 1). Furthermore, comparing sample replicates and samples taken in different years (Figure S3; years indicated on the abscissa) suggests that the ecology is steady, reproducible and deterministic, even though communities are complex and composed of many different species.

**Figure 3:**
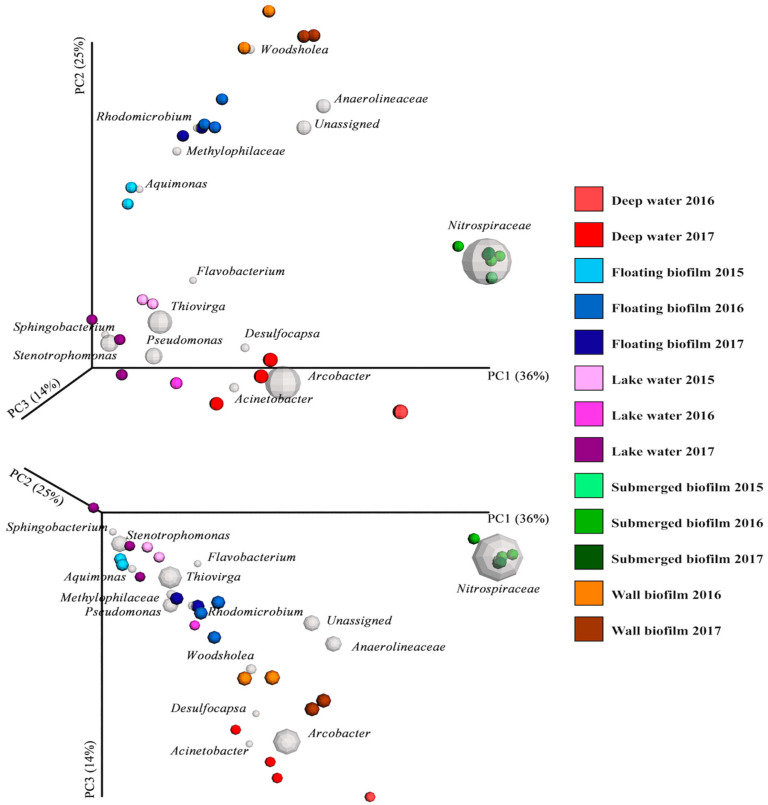
Three-dimensional principal coordinates biplot (weighted unifrac metrics) displayed from two angles. Variation between microbial communities in each sample is visualized by the placement of each sample (colored sphere) in the ordination field. The farther away these spheres are from each other, the more different their microbial profile is. Gray spheres represent the 15 most dominant OTUs (size represents relative abundance), and their position corresponds to the part of the ordination in which they are most strongly represented (i.e., in which samples these species are most abundant) and thus how they affect the ordination. The PC-axis 1 is the species composition that differentiates most between the samples. PC-axes 2 and 3 are the next most differentiating compositions. Samples plotted in the ordination are colored according to their sublocation in the cave (deep water, floating biofilm, lake water, submerged biofilm, wall biofilm), and the different sampling years are represented by variations of the base color, which refers to the colors given to each sublocation in Figure 1. The single deep-water 2016 sample is the orangelike sample that has the strongest PC1 character; the two 2016 wall-biofilm samples are the orangelike circles with the strongest PC2 character.

These observations are confirmed by a principal coordinates analysis (Figure 3), in which clear clusters are observed (the more similar the microbial communities of a sample are, the closer they occur in the plot). Accordingly, some gray spheres in Figure 3 are close to a cluster (specific species are the dominant members at different sublocations), suggesting that prevailing conditions per site favor certain species above others.

An adonis test (used to describe the strength and significance of a chosen variable on a distance matrix) gave a significant *p*-value (0.001) for the sublocations, with a correlation coefficient R^2^ (effect value) of 0.83, confirming that the microbial communities we observed are site-specific. Microbial community properties of the samples from the individual sublocations are discussed in detail in the Supplementary Materials (Section S1).

### 3.3. Mineral Microcosms

A variety of minerals were incubated in the deeper water layers (Figure 1) using the setup shown in Figure S1. All minerals were heat-sterilized prior to incubation. The different minerals and their chemical formulas are shown in Table 2. The minerals were selected on the basis of generating a diversity of environments, not because they corresponded to minerals found naturally in Movile Cave. By introducing different minerals into Movile Cave, we aimed to investigate whether differences in mineral composition would result in mineral-specific colonization patterns by microorganisms endemic to the cave waters and thus if certain minerals may contribute to the success (or failure) of colonization by microbial life.

**Figure 4:**
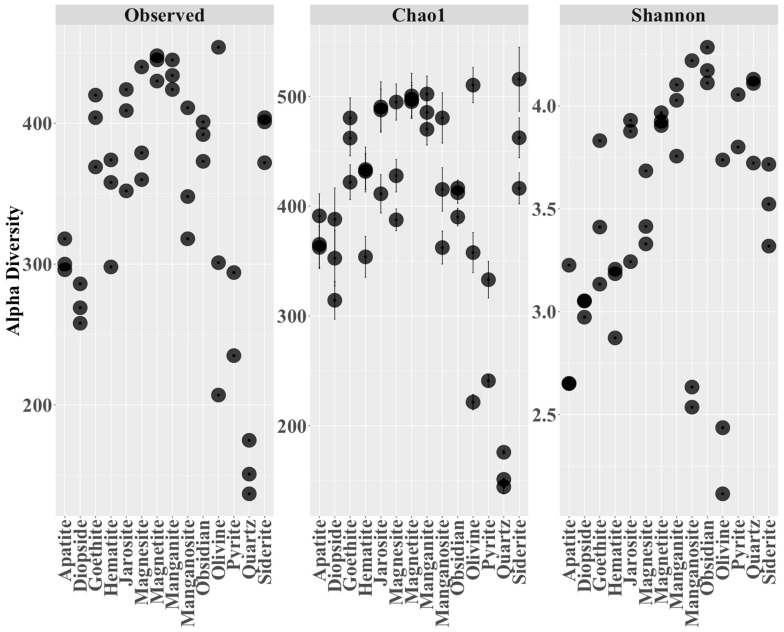
Microbial diversity of microcosms emerging on the 14 minerals introduced in triplicate in the cave’s deeper waters. Three alpha diversity metrics are shown: the observed number of species (‘Observed’), the predicted number of species based on sequencing depth (‘Chao1′) and the Shannon entropy focusing on how evenly the species are distributed (‘Shannon’). Error bars for the Chao1 index show the standard error of the estimated average richness of each sample (estimate_richness function in Phyloseq package). Samples were rarefied to an equal depth of 19,767 sequence reads for this analysis, and one of the pyrite triplicates was omitted because of its lower number of reads.

The DNA extracted from the minerals at the end of a one-year-long incubation was analyzed using Illumina 16S rRNA sequencing. With the resulting dataset, a 0.05% trimming step was performed, and mineral blanks were sequenced and subtracted from the dataset, leaving us with 2.8 million sequence reads and a total of 802 OTUs. Replicate samples were merged and visualized per mineral type. Many (>250) ‘species’ colonized the mineral matrices (Figure 4), with quartz (which was selected as a negative control because of the metabolic irrelevance of Si^4+^) constituting an expected exception to alpha diversity, for Chao1-predicted diversity but not per se for Shannon entropy.

**Figure 5:**
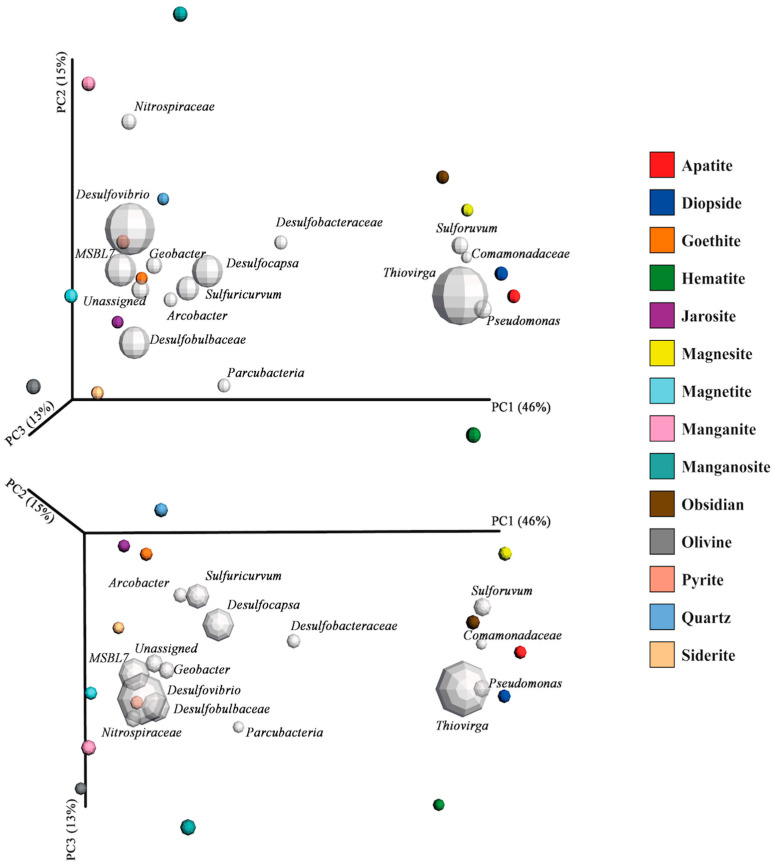
Three-dimensional principal coordinates analysis biplot (weighted unifrac metrics) of the microcosm microbiology displayed from two angles. Variation between microbial communities in mineral types is represented by the position of the sample (colored sphere) in the ordination field. The farther away the spheres are from each other, the more different their microbial profile is. Gray spheres represent the 15 most dominant OTUs (size represents relative abundance), and their position represents in which part of the ordination they are most strongly represented (i.e., in which samples these species are most abundant) and thus how they affect the ordination. PC-axes (1–3) display the maximum amount of variation (variation in community structure between the samples) that can be achieved along that axis. The triplicates of the minerals (including pyrite triplicates) were merged in single samples using the mean species number to facilitate interpretation (see Supplementary Materials for ordination representing the triplicates, Figure S6; Figure S7 shows the results of the corresponding principal coordinates analysis of mineral triplicates and submerged-biofilm plus deep-water samples). Samples were rarefied to equal depth of 7500 reads.

A wide variety of different phylotypes was found within the mineral matrices (Figure 5; see Figures S5–S7 for more detail). Family members of *Desulfobulbacea* were among the most dominant, while in the surrounding deep water, the *Campylobacteraceae* family (*Arcobacter*) was by far the most dominant (Figures S3 and S5). Indeed, many species found within the minerals were not detected in the deep-water samples in which the microcosm setup was placed, or at any other cave site investigated (Supplementary Materials, see Tables S1 and S2 for more detail; Figure S3). This indicates that microbes may be missed by traditional sampling methods and only become detectable after some ‘enrichment’ procedure, here consisting of the year-long microcosm incubation in the cave. Many of the low-abundance species detected in this ‘enriched’ dataset were present on most of the different minerals (Supplementary Materials, Table S2). This explains why all the mineral communities had relatively high alpha diversity. Also here, at the deeper layers of water with low oxygen concentrations where the minerals were incubated, we found phylotypes ranging from anaerobic to aerobic. This suggests that at least at some locations, sufficient oxygen seeps through to the deeper cave waters and sustains at least microaerobic respiration to a certain degree (see also Figure S2).

Focusing on the dominant taxa colonizing the different minerals (Figure S5), we found clear differences between minerals, while replicates of each mineral type were mostly similar (Supplementary Materials, Figure S6). The near absence of some species on some of the minerals, while being the dominant member on other minerals, indicates a strong preference of various species for (or dislike of) specific mineral types. This demonstrates strong adaptability of the local microbial ecosystems in the cave. This was largely confirmed by a principal coordinates analysis (Figure 5) in which two main clusters took form. The position of the cluster (magnesite, obsidian, apatite, diopside and to a lesser extent hematite) on the right-hand side of the primary axis (PC1) was strongly influenced by aerobic phylotypes, of which two belong to sulfur oxidizers even though none of these minerals contain sulfur. The cluster at the left end of the primary axis centers on dominant phylotypes belonging to (facultative) anaerobic and microaerophilic microorganisms, of which most are related to sulfate reducers.

Apart from quartz, the minerals positioned at the left end of the primary axis in Figure 5 all possess redox-potential carriers in the form of either iron or manganese. The minerals containing manganese are further separated from the bulk of the iron-containing minerals, which are strongly mediated by members of the *Nitrospiraceae* family (the uncultured member that was also present in high abundance in the submerged-biofilm samples) (Figure 3 and Figure S3).

The core cluster on the right end did not contain minerals with redox potential except for hematite, which appeared to be something of an outlier in the ordination. As expected, obsidian also falls in this cluster because of its much lower content of redox-active components in comparison to the iron- or manganese-containing minerals.

Adonis tests were performed to determine which properties of the minerals could best explain the observed variation in the microbial communities (Table 3). These tests were conducted on the datasets containing the original triplicate samples (rarefied to equal depth). Indeed, the mineral type explained the observed variation very well (*p* = 0.001, R^2^ = 0.79), confirming that the shaping of microbial communities was heavily influenced by the type of mineral.

By grouping the minerals by parts of their major components according to their chemical formula (Table 2), we aimed to test whether specific parts of the minerals were of relevance to microbial growth. From these analyses, obsidian was excluded. Many of the mineral components had significant *p*-values in adonis tests (Table 3). The only grouped mineral properties that showed relatively high R^2^ values and passed the significance tests were ‘iron’ and ‘redox potential’. This observation matches well with the clusters that make up the principal coordinates analysis, which seems most heavily influenced by the absence or presence of redox groups in the minerals. It is noteworthy that only the element sulfur and the carbonates had insignificant (>0.05) *p*-values in the adonis test, which may be related to the high concentrations of sulfur varieties and carbonates (due to limestone dissolution) in the cave waters.

In conclusion, the microcosm experiments showed that in the cave, the local mineral composition can determine the microbial community composition. Whether or not the mineral is redox-active (through Fe or Mn) is a major determinant, but otherwise, each mineral appears to offer a specific niche, probably determined by its more precise chemical composition and/or spatial structure.

## 4. Discussion

In the Supplementary Materials, our results are discussed in detail (Section S2). Here, we discuss the most important findings.

### 4.1. General Findings and Limitations of Our Methodology

Our most important finding is perhaps that even in Movile Cave, ‘Everything is everywhere but the environment selects’ [30]. ‘The environments’ even correspond to sublocations within the cave. Substantial diversity occurs at almost every location in the cave. The (apparent) diversity extends to rRNA sequences that are homologous to potential photoautotrophs. This may appear inconsistent with the isolation and darkness of the cave environment [1]. The observation may be explained by the fact that homology with photoautotrophs at the level or rRNA does not necessarily reflect the presence of photoautotrophic organisms in the cave: organisms in Movile Cave may share an evolutionary origin with photoautotrophs outside the cave. Alternatively, there may have been a failure of the seclusion of Movile Cave in recent history.

The microbial ecosystems also differ greatly between sublocations in the same cave. Many local communities contained phylotypes that appeared inconsistent with one another, such as aerobes and anaerobes. Further microheterogeneity of the conditions at the sublocations, such as in terms of (micro)aerobic and anaerobic pockets, facultative phylotypes (such as in facultative anaerobes) and evolutionary change in function of parts of genes may provide explanations of the diversity in the microbial ecosystems.

The methodology we used in the present paper addresses function only indirectly, i.e., through organism homology as inferred from 16S rRNA sequences. To establish function more definitively, one would not only have to identify the genes responsible for the processes inferred but also prove the activity of the corresponding enzymes and even measure pathway fluxes using isotope labelling. Since there are thousands of genes involved in each of hundreds of organisms, this approach would be served by having some clear hypotheses. As illustrated in the next section, such hypotheses may be inspired by a first bird’s-eye view, as provided for by our methodology.

### 4.2. Potential of the Endemic Microbial Communities

In the Supplementary Materials, we discuss in detail how Movile Cave is home to DNA sequences that are homologous to 16S rRNA sequences of organisms that together have a substantial metabolic potential: the corresponding total metabolic capacities may more than suffice to catalyze Gibbs energy harvest from redox reactions as well as the three most important element cycles, i.e., those of carbon, nitrogen and sulfur. With the caveat that this is highly speculative, we here hypothesize possible elemental cycles with some of the organisms that might catalyze them. Most of these example organisms could perform multiple steps (e.g., *Desulfobulbaceae* members can fully reduce sulfates to hydrogen sulfide by themselves).

For the C-cycle:
Fe^II^ + CO_2_ → [*Leptospirillum*] → Fe^III^ + organic carbon (or S + CO_2_ → [*Thiobacillus*] → SO_4_^2−^ + organic carbon)                    (1)Organic carbon + SO_4_^2−^ → [*Thermodesulfofibrio*]→ CO_2_ + S^2−^,with as feeding reaction CH_4_ + O_2_ → [*Methanotrophs* (e.g., *Methylococcales, Methylococcaceae)*] → organic carbon + CO_2_


An N-cycle may be catalyzed as follows:
NO → [*Pseudomonas*] →N_2_O → [*Pseudomonas*] → N_2_ → [*Azospirillum*] → NH_4_^+^ →                    (2)[COMAMMOX; *Nitrospira,* Beijerinckiaceae] → NO_2_^−^ → [COMAMMOX; *Nitrospira*,*Beijerinckiaceae*] → NO_3_^−^ → [ANME] → NO_2_^−^ → [ANME] →NO


(ANME = anaerobic methane oxidizers; COMAMMOX = certain members of the *nitrospira* genus.)

A sulfur cycle may also be sketched:
S→ [Thiobacillus; Sulfuricurvumkujinese]→ SO_4_^2−^→ [Desulfobulbaceae] → SO_3_^2−^ →                    (3)[Desulfobulbaceae] →S^2−^ (H_2_S) → [Arcobacter sulfidicus, Thiovirga] → S


In parallel, *desulfurellaceae* can reduce sulfur to sulfide:
H_2_ + S → [*Desulfurellaceae*] → CO_2_ + H_2_S                              (4)

The above reactions constitute hypotheses that require strong experimental support before being considered real. Our formulation in this explicit manner is meant to support the experimental testing.

In addition, a plethora of electron transfer (redox) reactions could be catalyzed by the organisms, involving iron, manganese, hydrogen, carbon, sulfur and nitrogen in various oxidation states. Ultimate sources of electrons and of the element sulfur may be the hydrogen sulfide and methane flowing into the cave, whereas the molecular oxygen in the air provides ultimate oxidation potential. The nitrogen should derive from the molecular nitrogen in the air or from the ammonia in the groundwater that flows into the cave.

The various organisms we mentioned in the above sketches may not all occur in the same niches; exchange of gaseous (N_2_, NO, N_2_O, NH_3_, O_2_, CO_2_, H_2_S) substances and aqueous solutes (SO_4_^2−^, SO_3_^2−^, S^2−^, NO_2_^−^, NO_3_^−^, HCO_3_^−^) and sulfur floating on the water may take care of the metabolic communication between the niches. At present, the cycles sketched here can only serve as hypotheses for further work. Such work may use genomewide metabolic maps and flux balance analysis to identify corresponding pathways and from there infer the relevant ‘metabolic’ genes, which one could then search for in the metagenome sequences determined for the relevant sublocation in Movile Cave. Pathways and organisms identified, one could then try to measure the enzyme activities and pathway fluxes experimentally.

### 4.3. Explanations of the Beta Diversity in the Endemic Microbial Communities

The differences between the communities at different sublocations may arise from the spatial variation in atmospheric composition, solute concentrations and solid substrates. The latter may be inferred from the results of the microcosm experiment. Niche differentiation was also observed for the sulfur-oxidizing communities in the Frasassi caves in Italy, where their distribution depends on the sulfide-to-oxygen ratio [31]. In Lower Kane cave, a sulfidic cave system in Wyoming (USA), the abundance and diversity of (especially) *Epsilonproteobacteria* changed according to sulfide and oxygen concentrations in the water [32]. Sulfur-to-oxygen ratios may be of similar importance for Movile Cave. Simultaneous sampling in Movile Cave sublocations for chemical and biological analysis may lead to stronger correlations, especially when functional gene analysis is included.

### 4.4. Comparison to Previous Findings on Microbial Communities in Movile Cave

The present study differs from earlier studies that focused on the microbial diversity in Movile Cave [1,6]: It is the first study to address many different locations within the cave, considering the diversity between them and this at three time points. In addition, the present study incubated microcosms of a wide range of minerals in the cave itself. Our next-generation sequencing techniques (as opposed to the clone libraries mostly used hitherto), thereby detected roughly six times more OTUs than the earlier studies did (results not shown). Our results reveal much greater diversity in Movile Cave than previously reported and, most importantly, reveal strong differences in community compositions between the various sublocations, which should therefore be considered as separate, specialized niches. Our results are not definitive with respect to the metametabolic processes that occur at various locations in the cave. Experiments administering isotope-doped substrates for growth at various locations in the cave followed by isotopic measurements of the resulting biomass will be more definitive on this count. This type of experiment focusing on methanotrophs and performed on added microcosms indeed revealed active assimilation into biomass [7].

### 4.5. The Effects of Minerals

The microcosm experiments led to three distinct observations. The most obvious finding might have been expected: the microbial communities arising on the newly incubated minerals differed from those in the immediate cave surroundings. This confirmed that ‘the environment selects’ should be interpreted in the sense of the material, not only biotic, environment [9,10,11,12,33,34].

The second observation was more of a surprise: many species on the incubated minerals were absent from the previously investigated sublocations in Movile Cave. Apparently, the cave is home to many microorganisms that occur at low abundance and can only be detected after enrichment through cultivation under conditions that are more favorable to them, such as on the minerals (see [6,35,36,37]). This suggests that even more organism types may be found in in situ cultivations when an even wider range of conditions like temperature, pH and redox-active substances is offered.

Our third major observation was that the chemical composition of the incubated minerals affected the distance matrix we used in the adonis tests, at least to the extent that this determined redox activity. As expected, the chemistry appeared to be important where it could matter most for the microorganisms: in the ability to provide oxidants or reductants for electron-transfer processes that could provide Gibbs energy for growth. That selective colonization of minerals depends on a variety of additional factors including accessibility, surface area, surface structure and pH was confirmed here by all minerals producing their own microecologies. The niches are defined by redox activity and even more by mineral-specific structure.

The effect of the mineralogy on biological systems seems to be more than the simple sum of parts, with the exciting implication that it may become possible to ‘fish’ for ecosystem types in diverse environments using these mineral microcosms.

## 5. Conclusions

Much is still unknown about subsurface life, and in our data, we have paradoxes that warrant further investigation: samples containing both strictly anaerobic as well as aerobic phylotypes; 16S rRNA homologous to phototrophs; and dominance of dedicated iron-reducing microorganisms on fully reduced iron minerals. The interactions taking place appear to be more complex than our 16S rRNA analysis can fully unravel. In a next step, functional gene analysis, specific target enrichment and isotope flux measurements should pinpoint the diversity in Movile Cave in terms of biochemical activities. Knowing how life operates in subterranean environments may also aid missions focusing on life detection in extraterrestrial subsurfaces.

## Figures and Tables

**Table 1:** The total number of OTUs (‘Total’), unique OTUs (‘Unique’) and percentage of unique OTUs (%) for each subsample location. All samples coming from the same sublocation (independent of sampling year) were merged into a single sample by using the mean abundances between the samples. These were compared to the combined samples from the rest of the cave. For these numbers, a nonrarefied (but trimmed) dataset was used.

Location	Total	Unique	%
Lake water	359	2	0.5
Deep water	424	0	0
Floating biofilm	734	317	43
Submerged biofilm	752	122	16
Wall biofilm	547	120	21

**Table 2:** Minerals incubated at the bottom of the deep-water section in the microcosm experiment (Figure 1, black star). The chemical formulas shown in this table were used as the definition for the composition of the mineral. * Obsidian is volcanic glass dominated by vitreous silicate phases. Other components of obsidian vary in abundance but often include smaller contributions of oxides of Al, K, Mg, Na, Fe and Mn.

Mineral	Chemical Formula
Apatite	Ca_5_(PO_4_)_3_(OH, F, Cl)
Diopside	MgCaSi_2_O_6_
Goethite	Fe^III^O(OH)
Hematite	Fe^(III)^_2_O_3_
Jarosite	Kfe^(III)^_3_(OH)_6_(SO_4_)_2_
Magnesite	MgCO_3_
Magnetite	Fe^II^Fe^III^_2_O_4_ (Fe_3_O_4_)
Manganite	Mn^(III)^O(OH)
Manganosite	Mn^(II)^O
Obsidian	SiO_2_ (65–85%) *
Olivine	(Mg^(II)^, Fe^(II)^)_2_ SiO_4_
Pyrite	Fe^(II)^S_2_
Quartz beads	SiO_2_
Siderite	Fe^(II)^CO_3_

**Table 3:** Adonis tests giving significance values (*p*) and effect sizes (R^2^) for the effect of the mineral type, redox potential and elemental components in the incubated minerals on the microbial variation observed in the ordination. For these analyses (except for mineral type), obsidian was excluded as an explanatory variable because of the uncertainty of its components. Italic font indicates that these components are only present in a single mineral and test results should therefore be interpreted with caution. Only the components with an * were positively tested for homogeneous dispersion within the sample groups. Tests were performed on the dataset rarefied to 19,767 sequence reads.

Component	*p*	R^2^
Mineral type	0.001	0.79 *
Fe (Fe^2+^, Fe^3+^)	0.001	0.16 *
Ca	0.001	0.16
Fe^2+^	0.001	0.14
Redox-active	0.002	0.13 *
Mn (Mn^2+^, Mn^3+^)	0.004	0.10
*Si*	*0.005*	*0.10*
*F*	*0.003*	*0.08*
Mn^2+^	0.015	0.07
*P*	*0.005*	*0.07*
*Cl*	*0.005*	*0.07*
Fe^3+^	0.031	0.064 *
Mg	0.03	0.056 *
Mn^3+^	0.03	0.06
S	0.10	0.04
CO_3_^2−^	0.5	0.02 *

## Data Availability

This Targeted Locus Study project has been deposited at DDBJ/ENA/GenBank under the accession KERY00000000, KERZ00000000. The version described in this paper is the first version, KERY01000000, KERZ01000000. Separate bioprojects were created for the microcosm experiment (PRJNA703821) and the experiment focusing on the endemic communities in the cave (PRJNA702640).

## Supplementary Materials

The following supporting information can be downloaded at: https://www.mdpi.com/article/10.3390/life13112120/s1: Supplementary Materials file with Figures S1–S7 included, Tables S1–S2 uploaded. References [38,39,40,41,42,43,44,45,46,47,48,49,50,51,52,53,54,55,56,57,58,59,60,61,62,63,64,65,66,67,68,69,70,71,72,73,74,75,76,77,78,79,80,81,82,83,84] are cited in the supplementary materials.
