# Supplementary material for: Microbial Ecosystems in Movile Cave: An Environment of Extreme Life
Source: Life (Basel). 2023 Oct 26;13(11):2120. doi: 10.3390/life13112120 (PMC10672346; doi:10.3390/life13112120)
Supplement: Supplementary file 1 [file life-13-02120-s001.zip › life-2666076-supplementary.pdf]

Supplementary Material

Microbial ecosystems in the Movile Cave: An environment of extreme life

Joost W. Aerts *et al.*

S1. Results on endemic microbial community properties at the individual sub-locations

Lake-Water samples varied most in terms of community profile between different sampling years. This is mainly due to the rise (and fall) of a few dominant community members (Figure S3). *Thiovirga* (making up 99% of the *Halothiobacillaceae* family in Figure S3), which were the dominant species in the 2015 samples, gradually declined in abundance until in 2017 they only made up a few percent of the community. *Flavobacterium* (making up 80% of the *Flavobacteriaceae* family in Figure S3) also largely disappeared after 2015. These two were replaced by *Pseudomonas* (100% of the Pseudomonadaceae), *Stenotrophomonas* (100% of the Xanthomonadaceae) and *Sphingobacterium* (100% of the *Sphingobacteriaceae*), which dominated the communities in 2017. The latter two were not noticeable during the first two sampling years. This sudden shift may relate to the prominence of a denser white biofilm in the cave passage connecting the Lake-Room to Air-Bell 1, which was becoming more visible from 2016 onward (not sampled for this study). This denser veil had been observed several years earlier as well and may be a periodic event that influences the communities in the surface waters. The first two years (2015 and 2016), the Lake-Water samples were dominated by aerobic sulfur oxidizers (e.g., *Thiovirga*), indicating that extensive sulfur oxidation occurred at the lake surface. For 2017 it was less obvious what the dominant microbial process at the surface water was, due to the lower contribution of *Thiovirga*. Sulfur oxidation may still have been dominant since certain members of the *Pseudomonas* genus and the *Xanthomonadaceae* family (dominant in 2017) are also capable of sulfur oxidation [38, 39, 40].

Deep-Water samples were only collected in 2016 and 2017, from the waters directly surrounding the place where the microcosm experiment (see below) had been installed in 2016. Samples of both years were dominated by an *Arcobacter* (99% of the Campylobacteriaceae in Figure S3) species, most likely *Arcobacter sulfidicus*, which is an obligate microaerobe that oxidizes sulfides, and an autotrophic producer of filamentous sulfur [41, 42]. This community member could be responsible for the thin white veil that was observed just underneath the water surface in the Lake-Room. Indeed, this *Arcobacter* species was also detected in appreciable numbers in the Lake-Waters and may be present in differing numbers along the oxygen gradient of the water column. *Desulfocapsa* (99% of the *Desulfobulbaceae* family in Figure S3, *Methylobacterium* (100% of *Methylobacteriaceae* family) and *Acinetobacter* (100% of the *Morraxellaceae*) are some of the other abundant community members in the Deep-Water samples. Also, in these samples *Pseudomonas* species became more abundant in 2017.

The Deep-Water communities represented a variety of metabolic potentials ranging from anaerobic to aerobic as well as sulfur-cycling, nitrification (including members of the *Beijerinckiaceae* family which are free living aerobic N_2 fixers often with methane- and other C-1 metabolizing capabilities [43].

Submerged-Biofilm samples were scraped off rocks collected from the bottom of the submerged passages and had a greyish color. Samples showed high alpha diversity (due to a large number of low abundance species) but remained highly similar over the different sampling years. The samples from 2017 showed somewhat higher species diversity than those from the previous years. However, every year communities were dominated by a single species, namely an uncultured member of the *Nitrospiraceae* family (100% of the *Nitrospiraceae* family in Figure S3). The *Nitrospiraceae* family is physiologically diverse and contains chemolithoautotrophic aerobic nitrite-oxidizing bacteria (*Nitrospira*), chemolithoautotrophic aerobic and acidophilic ferrous iron oxidizers (*Leptospirillum*), and anaerobic, thermophilic, chemoorganoheterotrophic or hydrogenotrophic sulfate reducers (*Thermodesulfovibrio*). Family members have been found in a wide range of habitats [44]. The *Nitrospira* genus is a predominant nitrite-oxidizer in nature, mediating the second step in the nitrification cycle (nitrite to nitrate) and is thus important for biochemical nitrogen cycling. Moreover, certain members of this genus are able to complete all the steps of the nitrification process (i.e., $NH_4^+ \rightarrow NO_2^- \rightarrow NO_3^-$) on their own (collectively called COMAMMOX bacteria; see Eq. 2 in the Discussion section) [45, 46], which would be a desirable trait in a secluded cave environment. Members of *Leptospirillum* are important iron oxidizers, often found in mine drainage residues due to their role in mineral ore bioleaching [47]. *Thermodesulfovibrio* are found in anaerobic habitats, where they contribute to the degradation of organic compounds, making them indirectly responsible for the production of methane [48]. It seems that a new member of the *Nitrospiraceae* family is present in the *Movile Cave* ecosystem since it cannot be placed within these existing genera based on its phylogeny.

Further species detected in the Submerged-Biofilm suggest (at least locally) anoxic conditions, since strict anaerobes are more prominent (e.g., Desulfobulbaceae, Anaerolineaceae). A member of the deltaproteobacterial "*Candidatus* Acidulodesulfobacterales", i.e. sva 0485, was also prominent, which are known as anaerobic sulfate reducers [49]. We also detected Deferribactereaceae, which are known to engage in anaerobic respirations using manganese, iron or nitrate as electron acceptor. However, the presence of microaerobic (i.e., *Arcobacter, Thermoflexus*) and facultative anaerobic (*Aeromonas*) species suggests that some oxygen was still present in the deeper layers of the water, which was confirmed by our oxygen measurements throughout the water column in the Lake-Room. Obligate anaerobes are able to create anoxic pockets. A similar mechanism could explain how aerobes and anaerobes coexist.

The few Archaea found in the cave (0.25% of the total sequence reads, not shown in figures), were detected mainly in the Submerged-Biofilm. Most notable were two members of the ANME (anaerobic methane oxidizers) family. These family members perform the anaerobic oxidation of methane coupled with the reduction of sulfate, nitrate or nitrite provided by a Bacterial 'symbiotic' counterpart [50, 51].

Some other Archaea were detected as well, including members of the Deep Sea Hydrothermal Vent Group (DHVEG). These Archaea were first discovered living on reduced gases arising from hydrothermal vents on the ocean floors. Their presence here confirms that they may be widespread in non-oceanic subsurface environments as well.

Floating-Biofilm sample analysis revealed the highest species diversity and the most complex communities. These samples were collected from the microbial mats floating on the water surface in Air-Bell 2 (Figure 1), where the local atmosphere is reduced in oxygen (7-10%), while enriched in CO_2 (2-3%) and CH_4 (1-2%). In these samples we observe more evenness in the species distribution with few to none strongly dominating community members but many in lower abundance (Figure 2, Figure S3). Accordingly, the metabolic potential appears very diverse. If these homologies are indicative of cell functioning, all the major elemental cycling metabolisms should be present, thereby creating a self-sustaining ecosystem. Part of the species composition may reflect the lack of oxygen expected in biofilms where anaerobe pockets arise due to oxygen consumption.

The more pronounced species include a member of the genus *Sulfuricurvum* (making up 10-50% of the *Helicobacteriaceae* family in Figure S3), and of which the type species (*Sulfuricurvum kujinese*) is a chemolithoautotrophic, facultatively anaerobic sulfur oxidizer [52]. We found *Methylophilaceae* family members, which are known to metabolize C_1 compounds such as methyl-amines and methanol (but not methane) as a sole source of carbon and Gibbs energy source. Methane-oxidizers were present (e.g. *Methylomonas and Methyloparacoccus*, making up the largest part of the *Methylococcaceae* family in Figure S3). The *Azospirillum* species (making up a large part of the *Rhodospirillaceae* family in Figure S3), also detected in higher numbers, are microaerophillic nitrogen fixers. *Paludibacter* (an anaerobic chemoorganotroph from the Solidibactereaceae family), *Thiobacillus* (a sulfur-oxidizer from the *Hydrogenophillaceae*), and *Hydrogenophaga* (a hydrogen-oxidizers from the Comamonadaceae family) occurred as well. Intriguingly, several phototrophic phylotypes were detected in relatively high numbers (e.g., *Rhodomicrobium*, *Chlorobiales*).

The microbial profiles of the different sample years are remarkably similar even though the exact location from where the biofilm samples were taken differed within and between years (biofilms cover the water in the Air-Bell making it impossible to sample from the exact same location). At the same time, the biofilms differed drastically from the other cave locations in terms of microbial composition and show the highest number of OTUs that are unique for this sub-environment.

Wall-Biofilm samples showed a picture similar to that of the Floating-Biofilm samples with respect to the high diversity and evenness of the species distribution (Figures 2, S3). The biofilm samples showed strong consistency in species profile over the course of the two sampling years. However, even though these biofilms covered the dry walls in the same Air-Bell as the Floating-Biofilms did, their communities were highly different in terms of species composition. Uncultured *Methylococcales* and *Methylococcaceae* family members were present, which are generally considered methanotrophs. Since elevated levels of methane were present in the Air-Bell, these findings support the selective effect of the environment.

The presence of a variety of methanotrophic phylotypes in Air-Bell 2 also supports an important role to methane oxidizers in the sustenance of the *Movile Cave* ecosystem [13]. Also, relatively high numbers of *Nitrospira* (*Nitrospiraceae* family) species were detected. All known members of this genus are capable of nitrite-oxidation and some have even been shown to perform the complete nitrification process (i.e., ammonium oxidation to nitrate) on their own (COMAMMOX) [46]. The dominant *Woodsholea* species in these samples may not be a particularly exciting marine bacterium. However, its dominance in this environment suggests that this particular strain may have developed some interesting characteristics.

The family *Desulfurellaceae*, which was also detected in appreciable numbers, contains the genera *Desulfurella* and *Hippea*. Seven valid species exist within this family, all strictly anaerobic and moderately thermophilic. Members of the family are mostly sulfur respiring, though *Desulfurella propionica* can use thiosulfate as alternative. During growth, organic substrates are fully oxidized in terms of their carbon, with CO_2 and H_2S as end products. Most of these species are capable of growth with hydrogen as electron donor.

Also, in these biofilms, much metabolic potentials coexists thereby, i.e., from aerobic to strictly anaerobic, and from heterotrophic to chemolithoautotrophic. The 16S phylogeny suggests that the most important elemental cycles (N, C, and S; see section 4.2) may all be possible in this sub-environment.

S.2. Supplementary discussion

S2.1 General findings and limitations of our methods

The difference between species found at the different cave locations could be due to different initial seeding and lack of communication between the locations, or due to a plethora of microbial infections, with the local environment selecting ('Everything is everywhere but the environment selects' [30]. Since the submerged passages connect the different areas of the cave and incubation of minerals led to new microbial populations different both from their surrounding water and from other cave locations, the latter appears more likely.

The potential for different metabolic processes (aerobic, anaerobic, S, N, C, C-1 (CH_4), Fe, Mn metabolism (chemo, organo, litho, hetero, auto)) was often observed within the same microbial communities. This mix of phylotypes was most extreme in the biofilm samples. Biofilms could be shaped in ways that anaerobic pockets exist next to oxygenated zones, as observed in biofilms in Lower Kane Cave (Wyoming, USA) [53, 54]. It may be necessary to zoom in further into these sub-environments to distinguish spatially between the microbial processes. Environmental and/or physical variation may exist on the millimeter scale.

Facultative switching between metabolisms, or evolutionary divergence between function and 16S phylogeny, could explain these opposing mixed phylotypes further. Functional genomics should shed more light on the biochemical functions and may help in revealing the role of the large fraction of unidentified/uncultured species that we detected (mainly in the biofilm samples).

The same holds true for the phototrophic phylotypes we found. Since no light reaches this environment, phototrophic these phylotypes cannot be indicative for their function. However, certain members of the *Chlorobiales* order have been found before in multiple places that are nearly (or completely) devoid of light, including a large population at 100 meters depth in the Black Sea [55]. Phototrophic phylotypes have also been found in the *Frasassi* caves (Italy) as well as in Lower Kane Cave (Wyoming, USA), which are similar to *Movile Cave* in terms of sulfidic input [56]. Even 'strict' phototrophs are able to switch metabolism when circumstances demand it [57]. This 'species-fluidity' may be a widespread phenomenon and demands caution in all eco-genomic analyses.

S2.2 Potential of the endemic microbial communities

Primary Gibbs energy provision has previously been reported to derive from sulfur-cycling [58, 59], although carbon (i.e., methane and other C-1 compounds), whilst nitrogen-cycling [6, 7, 60] also contributed significantly. In our sequence data, phylotypes capable of performing the metabolic steps needed in all these cycles, abounded.

Of special note was the apparent abundance of a variety of nitrogen-fixers such as members of the *Rhizobiales*, *Nitrospira* and the free-living *Azospirillum*, which can convert atmospheric nitrogen gas into biologically accessible forms [61]. The presence of these phylotypes showed that the important steps in nitrification are all represented in this ecosystem, and that members likely to be capable of these steps are more abundant than previously shown (see also Eq. 2 in section 4.2). The same goes for the sulfur cycle for which a vast number of species were found for both the sulfur oxidation steps (i.e., *Thiovirga*, *Arcobacter*) and the sulfur reduction steps (i.e., *Desulfobulbaceae*, *Desulfovibrio*). A variety of known methane oxidizers were also apparent in our sequence data (i.e., *Methylomonas*, *Methyloparacoccus*, ANME *Archeae*) as well as other C-1 metabolizing phylotypes (*Methylophilaceae*). This broad microbial support for all these major biological pathways may explain in part why microbial life flourishes in this extreme environment.

By far the majority of the phylotypes belonged to chemoautotrophs, which was expected due to their vital role in the primary production of biomass in the absence of photosynthesis and input of organics from the surface. Chemoautotrophic productivity was indeed two orders of magnitude higher than heterotrophic productivities in the Floating-Biofilms in Air-Bell 2 [36].

Based on our data, we suggest that the previously proposed cycling of S, C and N may well occur within each location as well as between the different locations in the cave. Oxidized compounds (i.e., sulfates and nitrates) can serve as alternative electron acceptors in areas where oxygen is scarce (i.e., the deeper waters). Due to continuous supply of reduced compounds by the mesothermal waters and the interconnection between most of the different areas in the cave via the submerged passages this cycle can be retained and replenished throughout the different cave areas.

S2.3 Explanations of the beta diversity in the endemic microbial communities

The differences between the communities at different sub-locations appear consequential to the differences in local conditions such as atmospheric composition, concentrations of oxygen or sulfur compounds in the water, or the presence of different minerals (the latter may be inferred from the results of the microcosm experiment). Although it has long been thought (also on the basis of experimental evidence) that deeper assume waters in *Movile Cave* are anoxic [8], we here showed with our oxygen measurements through the water column that at least at some positions, lower parts of the water may well contain oxygen, albeit at low concentrations. The low partial pressures are in the range of the K_M of terminal oxidases ((sub)micromolar) [29, 62]. As we found oxygen levels at the bottom of the water around 0.5 micromolar, microbes with high affinity terminal oxidases (or operating at lower protonmotive force [63] may be present at lower abundance in the deeper waters. The observed oxygen gradient throughout the water column may be paralleled by species profiles influenced by the K_M of their terminal oxidases. This correlates well with the phylotypes we found in the cave waters (which were often a mix of aerobes and anaerobes) and it also matches with a recent study which suggested the presence of microaerobic methylotrophs in supposedly anoxic sediments [64]. However, anaerobic zones likely exist due to both the metabolic action of high numbers of sulfur- and methane oxidizers and the influx of H_2S into the cave, which may be stronger at some places than at others, and cause abiotic reduction of dioxygen and sulfate formation. Rohwerder *et al.* [58] measured an up to 40-fold variation in sulfate concentrations between different sub-environments in the cave. This could both affect local biology and be an effect of local biology.

In our sequence data we see how the unique atmosphere in Air-Bell 2 (low O_2, high CH_4 and CO_2) correlates with higher abundances of species that should be expected to profit from that environment (e.g., methanotrophs). The deeper waters, where O_2 is scarcer, are accordingly host to a higher number of anaerobic microbes as opposed to the surface waters in the Lake-Room, which were dominated by (aerobic) sulfur oxidizers.

S2.4 Comparison to previous findings on microbial communities in *Movile Cave*

Most previous studies focused on the floating biofilms in Air-Bell 2, with the exceptions of a study in 2018 targeting methylotrophs in the sediments of the submerged passages [64], and a study that described a new *Thiovulum* species in the lake waters [65].

Our analysis of the Floating-Biofilms in the Air-Bells roughly revealed the same pattern as the previous studies that focused on these samples: Proteobacteria clearly dominate the biofilms [6, 36]. However, Chen *et al.* [6] showed especially high numbers of Gamma- and Betaproteobacteria, while our analysis revealed more or less equal relative abundances for Delta- and Alphaproteobacteria (and to a slightly lesser degree Epsilonproteobacteria), making it more similar to the profile described by Porter et al. [36]. We found a much higher contribution of Alphaproteobacteria. Furthermore, both quoted studies found abundant *Bacteriodetes*, while *Planctomycetes*, *Nitrospira*, *Firmicutes*, *Spirocheta* and *Verrucomicrobia* were detected in lower numbers.

S2.5 The effects of minerals

The observation that a significant fraction of the species found on the incubated minerals were absent from the previously investigated sub-locations in Movile Cave, suggests that there are many more species present in the cave waters, albeit in low numbers. This is significant since it confirms that under standard sampling strategies, the microbial diversity is underestimated [35, 37] and indicates that there may be many more micro-niches to explore in *Movile Cave*. These organisms may only become detectable after cultivating them by providing the right conditions. Also, earlier studies of *Movile Cave* were only able to identify certain species after cultivation experiments in the laboratory [6, 36]. We showed here that low abundance microbes could also become more dominant (and thus more easily detectable) by incubating a microcosm, *i.e.,* by giving them a site to adhere to and/or to profit from. Bacterial adhesion is the first step in a cascade of processes that can lead to surface colonization and biofilm formation [66]. Although microbial profiles may change over longer incubation times, 'fast' (at the time scale of a year) accumulation of microbes and specific growth profiles were already observed for all minerals, indicating that microbes in the cave waters responded quickly to changes in their environment.

The two manganese containing minerals that were incubated (manganite and manganosite) formed a slightly separate sub-cluster in the Principal Coordinates Analysis and both minerals had significant contributions of an uncultured member of the *Nitrospiraceae* family, which was the dominant microorganism in the Submerged-Biofilms. Perhaps this microorganism is responsible for the manganese-oxidizing/reducing capabilities. Microbial oxidation and reduction of manganese minerals has been shown for a wide variety of microorganisms in oxygenated as well as anoxic environments [67] and could be of significance in *Movile Cave*. Black silts were observed at numerous locations in *Movile Cave*, in the soils near the water surface in the Lake-Room as well as in the sediments covering the floors of the submerged passages. Manganese oxides have a blackish appearance and could be components of these black silts.

Most incubated minerals containing iron or manganese groups showed relatively high abundances of taxa specializing in reducing inorganic compounds, such as sulfates, nitrates, iron and manganese, that can serve as (terminal) electron acceptors in the absence of oxygen (Figure S5). Members of the *Desulfobulbaceae*, *Desulfurivibrio*, *Desulfocapsa* and *Nitropsiraceae* were amongst the most dominant. Gibbs energy is probably obtained by catalyzing electron flow between reduced or oxidized compounds in the cave waters and the redox active groups in or from the minerals. However, it is not yet clear in which direction this flow goes. Nitrate or sulfate reduction might be coupled to the oxidation of iron or manganese in the minerals. Under anoxic conditions, iron can also serve as a terminal electron acceptor which can be coupled to the oxidation of sulfur compounds [68]. The blackened soils we observed in Movile Cave can also be conglomerates of iron-sulfides, which can be formed by microbial activity of sulfate-reducers [69, 70]. Further investigation of these silts should reveal more about their origin and could tell us which microbes are involved in their formation.

We also aimed to infer function from phylogeny by introducing specific minerals. However, the complex nature of the communities combined with the complex and diverse conditions in the cave often made multiple explanations possible even for fairly specific observations. For example, we found that *Geobacter* species predominantly colonized pyrite (1-2 factors of magnitude higher than any of the other minerals). *Geobacter* are generally known as iron reducers, while pyrite or iron persulfide (FeS_2) is a mineral composed of already reduced (ferrous) iron and persulfide (S_2^{2-}). However, in a study by Weber et al. [71] certain *Geobacter* species were shown to be capable of anaerobic Fe(II) oxidation coupled to the reduction of NO_3^- to NH_4^+. This process should fit well in the *Movile Cave* environment and could explain the presence of *Geobacter* there.

There were more uncertainties. Hematite, a mineral rich in Fe^{III}, was home to communities that did not fall within the 'redox cluster' seen in the Principal Coordinates Analysis. The placing of this mineral in the 'non-redox' cluster is strongly influenced by a high abundance of *Thiovirga* species, which were much less abundant on the other iron or manganese containing minerals. Also, higher numbers of *Thiomicrospira, Pseudomonas* and *Comamonadaceae* were observed in hematite, making its dominant species profile more similar to that of the minerals that do not contain redox active components. Quartz on the other hand showed a species profile more similar to minerals containing redox potential, even though it had much lower alpha diversity than all the other minerals. Obsidian contains much SiO_2 (up to 90%) and Al_2O_3 (around 10%) with lower contributions of K_2O, Na_2O and FeO [72, 73]. Since we did not possess detailed knowledge on the composition of this particular sample we excluded it as an explaining variable in the adonis tests. However, its placing in the ordination plot together with the non-iron containing minerals fits the understanding that the iron concentrations in obsidian are often only 1%.

S2.6 The microcosm experiments and the geology of Mars

The microcosm experiments employed a variety of minerals that have also been detected on Mars (see for more information Table 1). Iron oxides and hydroxides such as hematite and magnetite are largely responsible for the red 'rusty' color and, respectively, the magnetic properties of the Martian dust [74, 75]. Goethite, the hydrated precursor of hematite, which can only form in the presence of water, has also been detected on Mars, albeit it appears less common [76]. Jarosite, an iron-sulfate which also only forms in aqueous environments has been used as evidence that water once existed at Mars' *Meridiana Planum* [77]. Many of these jarosite-bearing deposits are thought to have formed via aqueous oxidation of pyrite [78]. Pyrite has also been reported to be present in low quantities in a mudstone in Yellowknife bay [79]. A variety of carbonates have now also been identified on Mars including magnesium (magnesite) and iron (siderite) carbonates [80, 81]. The magnesium-iron silicate olivine is widely present on Mars and is an indicator of arid conditions since it readily weathers under aqueous conditions [82]. Obsidian-like molten glass, which may have formed at impact events, was detected on several Martian sites [83].

Obsidian itself is predominantly composed of non-crystalline silicate and is formed by rapid cooling of volcanic viscous lava flows, resulting in the formation of a glassy rock with a chemical composition similar to granite and rhyolite [72]. Incorporated traces of other oxides can serve as a potential source of nutrients for bacteria, although the accessibility appears to be dependent on the weathered state of the volcanic glass [84]. Diopside is a common pyroxene mineral found on Mars containing traces of magnesium and calcium. Apatite, also present on Mars, contains a phosphate group, which could be an important source of phosphorus for microbes, as well as a fluoride ion, which is often toxic to microbial life, at higher concentrations. Properties of such minerals may have a strong effect on colonization by microbes.

S3. REFERENCES for supplementary material are included in the main manuscript

S4. Supplementary Tables

Table S1: uploaded, i.e., available as separate pdf in the supplementary material

OTU table showing microbial endemic diversity in the various sub-locations in Movile Cave. OTU reads are shown for the non-rarefied dataset after the subtraction of various positive and negative controls.

Table S2 uploaded, i.e., available as separate pdf in the supplementary material

OTU table showing microbial diversity observed in the various mineral triplicates resulting from the microcosm experiment. OTU reads are shown for the non-rarefied dataset after the subtraction of various positive and negative controls, including the blank controls for the different minerals.

S5. Supplementary Figures

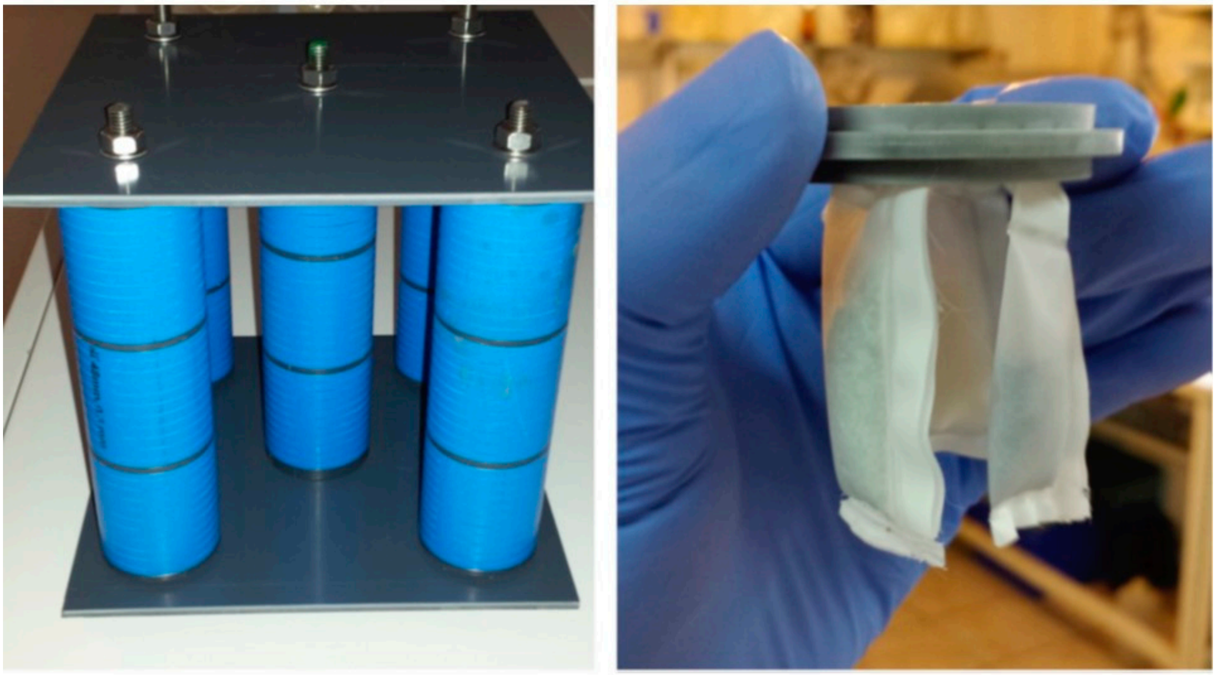

Figure S1

Device used for the microcosm incubation experiment. Each pillar consists of three separate compartments, in each of which three mineral bags could be stored, giving space for the total of 45 mineral bags. Slits in the compartments allowed cave water to freely flow through the device.

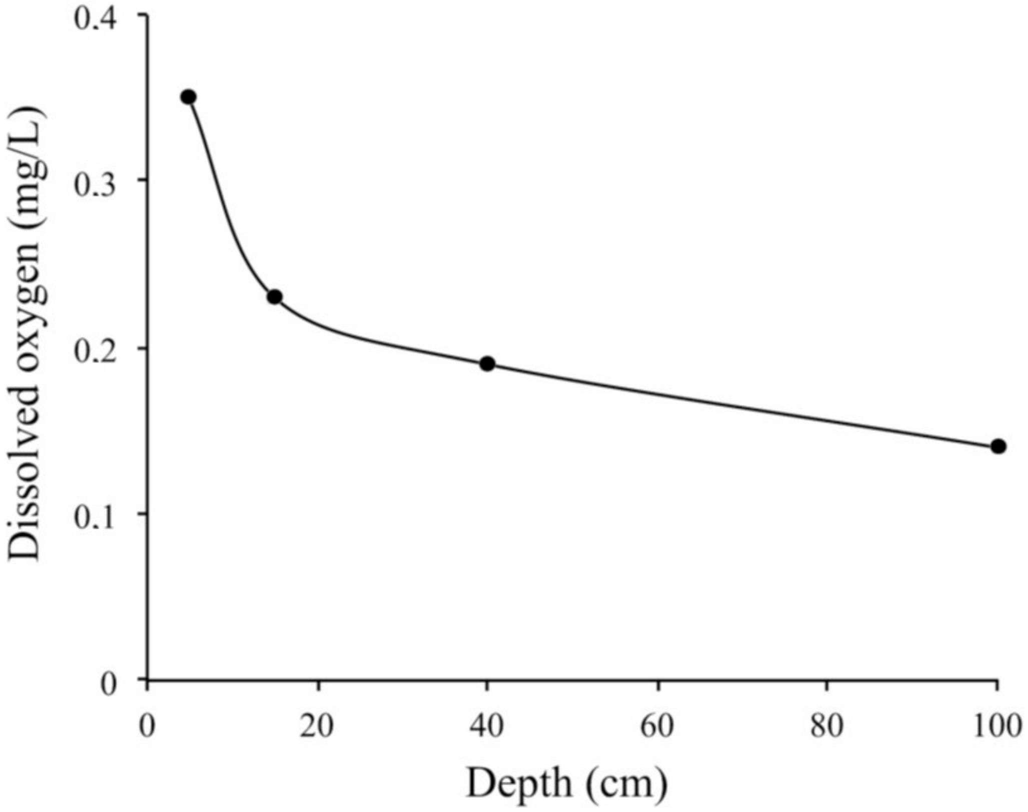

Figure S2

Dissolved oxygen in milligrams per liter as measured on site with an oxygen probe. These measurements were performed in 2017 in the waters of the Lake-Room. The 100 cm depth corresponds to the bottom of the lake. As a comparison, oxygen levels in fresh water equilibrated with air of 1 bar are around 9 mg/l (0.3 mM O_2) at a temperature of 20 °C.

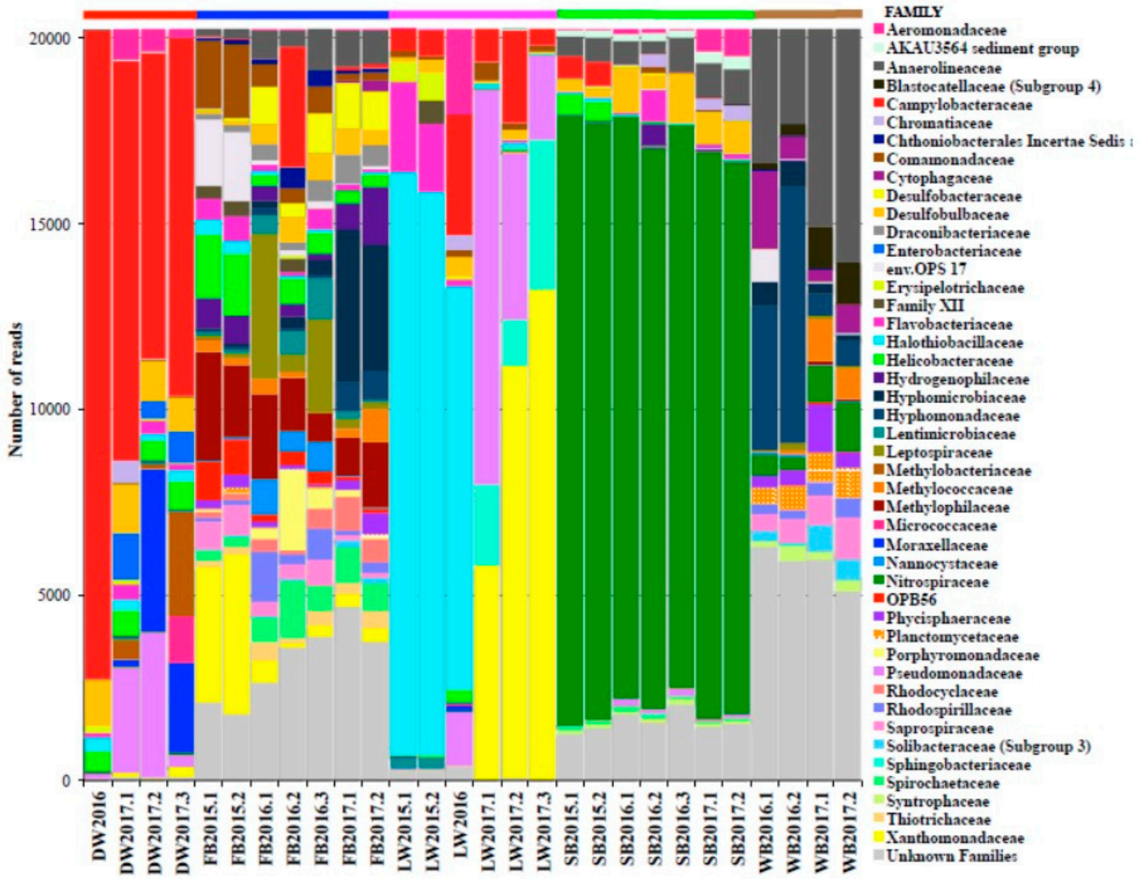

Figure S3

Bar plot depicting the microbial diversity displayed at the (prospective) family level (rarefied to equal depth). Only the partial dataset of the 50 most abundant family members is displayed in this figure. DW (Deep-Water), FB (Floating-Biofilm), LW (Lake- Water), SB (Submerged-Biofilm), WB (Wall-Biofilm) (2015-2017) refer to the cave locations and sampling years. The colored bars above the plot also indicate the sampling sites (red (DW), blue (FB), pink (LW), green (SB) and brown (WB) respectively, which correspond to the locations shown by the circles of the corresponding colors in Figure 1. In light grey the unknown families are shown, which comprise the total of taxa that could not be not assigned at the family level.

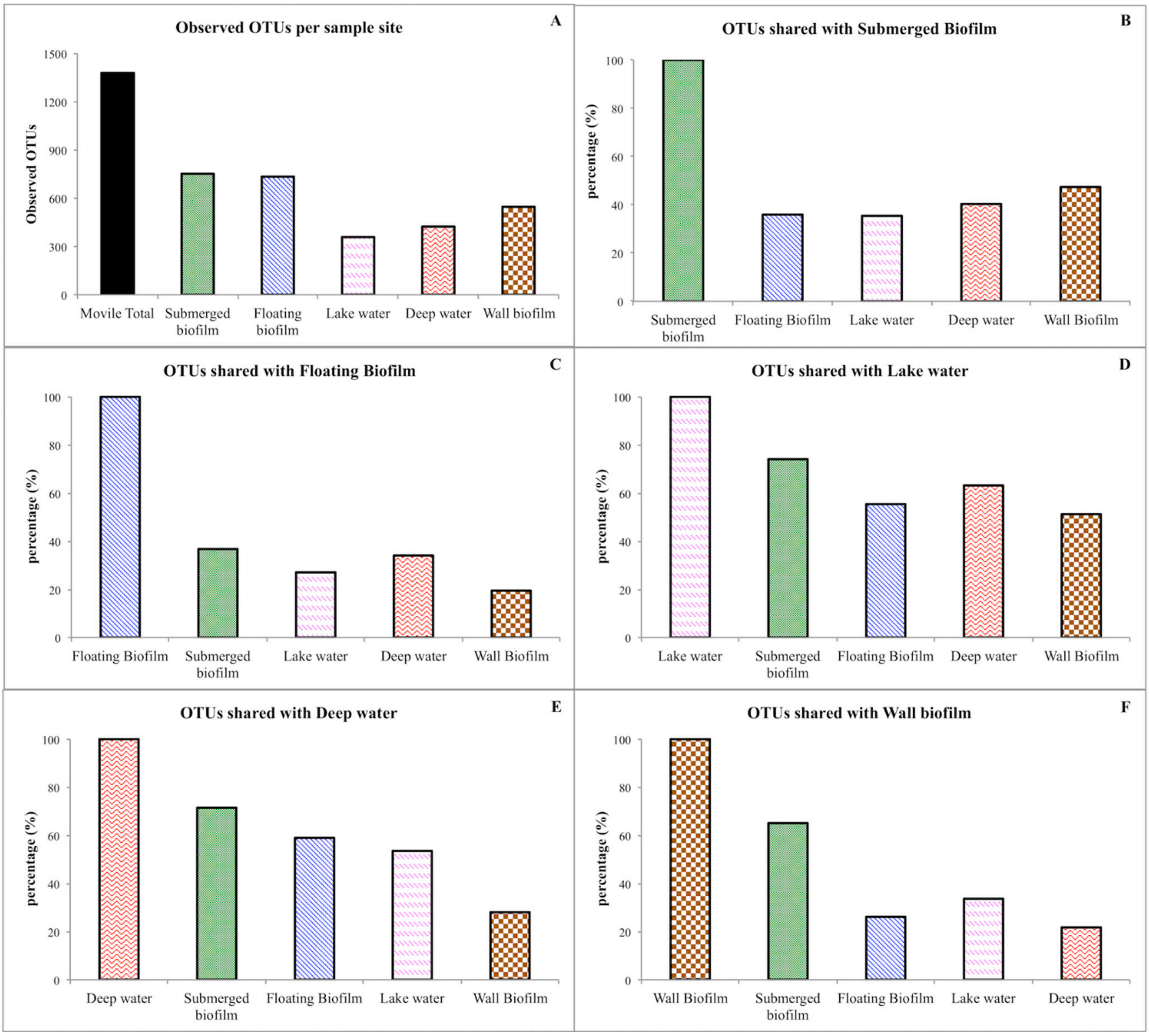

Figure S4

Overview of the number of species (OTUs) per cave location as compared to the total number of species in the dataset (A) and the number of species that the different sub-locations within Movile Cave had in common (B-F). Shared species are given in percentages of the total number of species of the sample site that they are compared with (which is always at the left side on the abscissa). For example, in Figure B, the Floating-Biofilm samples contain 35 % of the species that are observed in the Submerged-Biofilm samples. Colors of the bars refer to the colors given to each sub-location in Figure 1.

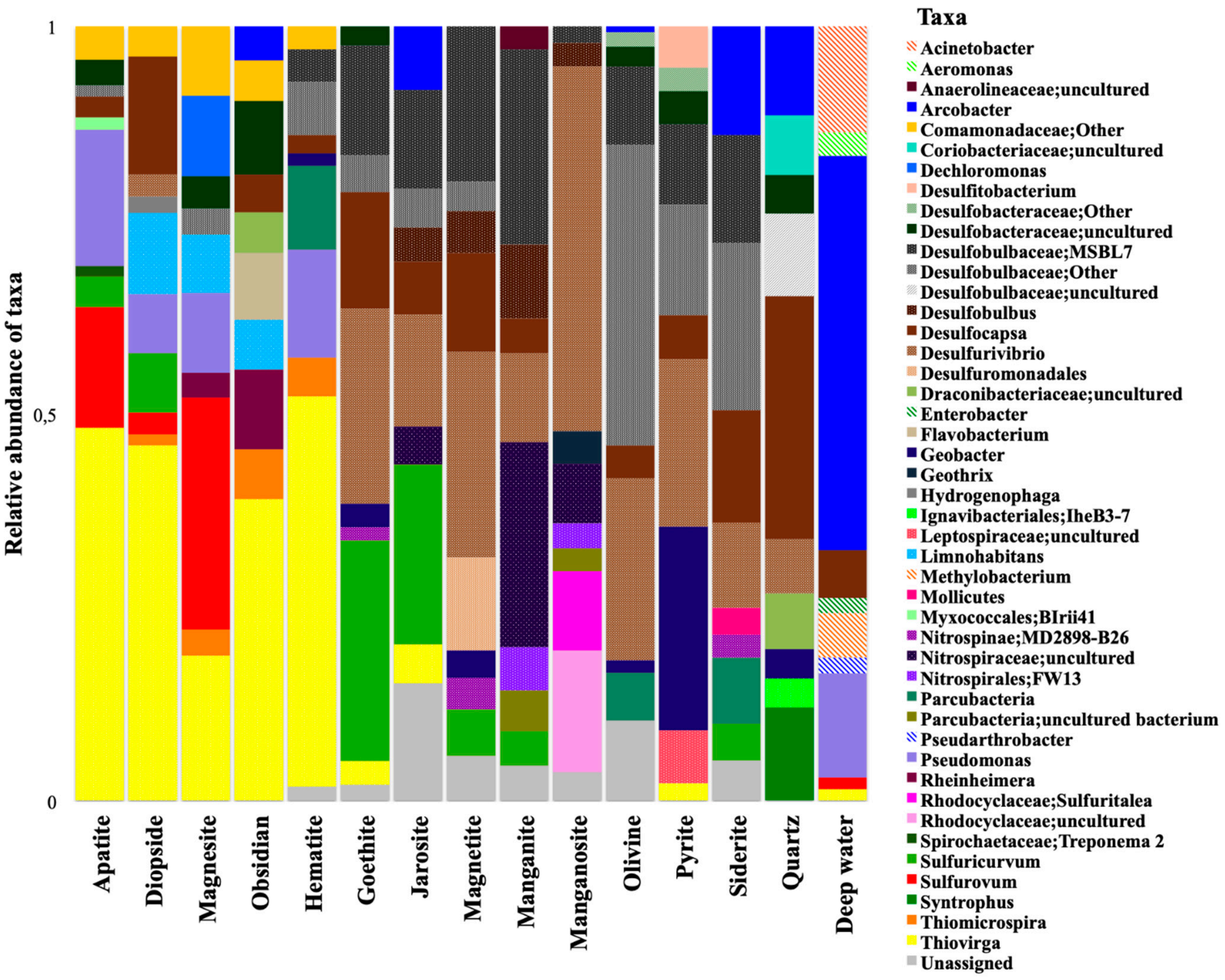

Figure S5

Stacked barplot depicting the 10 most abundant taxa for each incubated mineral type of the various microcosms. Triplicates (duplicates for pyrite) were merged into a single sample (mean) in QIIME to facilitate interpretation. On the far right the top 10 taxa are plotted from the merged Deep-Water (in which the microcosms had been placed) samples from 2017 for comparison to the mineral samples.

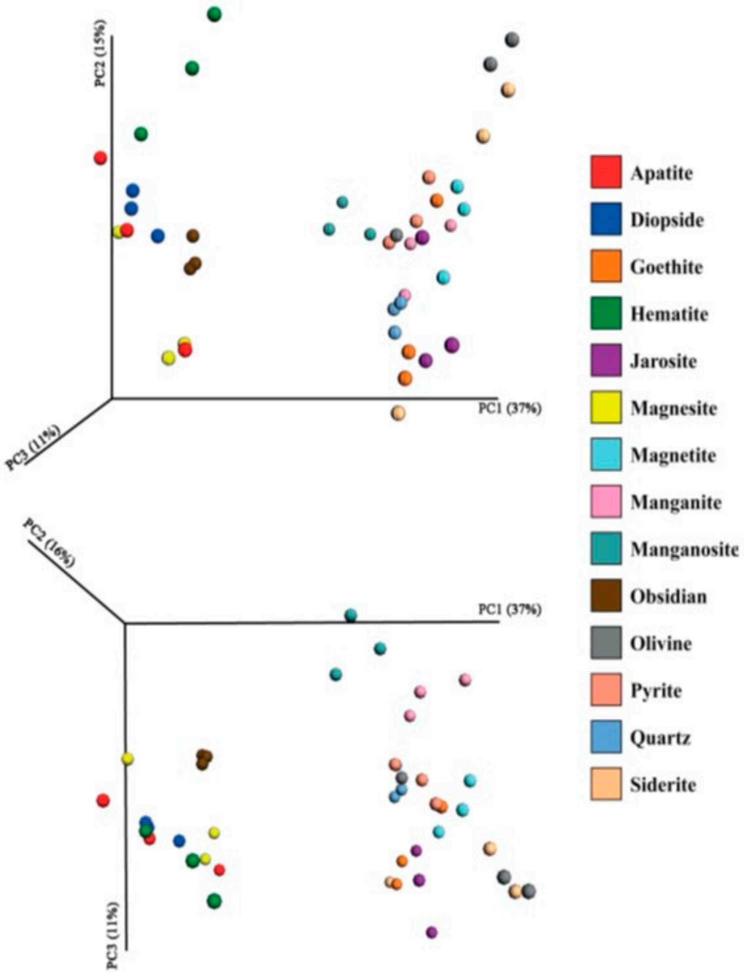

Figure S6

Principal Coordinates Analysis visualizing the triplicates from the incubated minerals in the microcosm experiment. Weighted unifrac metrics were used and the ordination is displayed as seen from two angles. Samples were rarefied to an equal depth of 7500 reads.

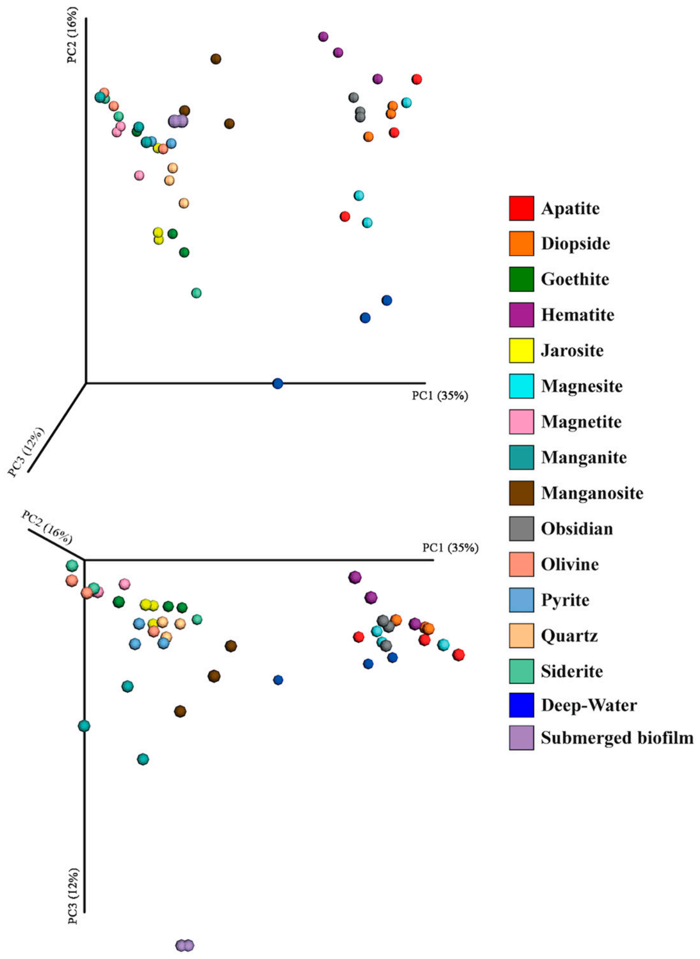

Figure S7

Principal Coordinates analysis visualizing the triplicates from the incubated minerals from the microcosm experiment as well as the 2017 samples from the submerged-biofilms and the deep-water samples, i.e. the two cave samples closest in space to the microcosms. Weighted unifrac metrics were used and the ordination is displayed as seen from two angles. Samples were rarefied to an equal depth of 7500 reads. It may be noted that the color coding is mostly different from that in Fig. S6 and that the sorting is similar except that the PC1 distribution is inversed.
